# Energy-Efficient Sensing in Wireless Sensor Networks Using Compressed Sensing

**DOI:** 10.3390/s140202822

**Published:** 2014-02-12

**Authors:** Mohammad Abdur Razzaque, Simon Dobson

**Affiliations:** 1 Faculty of Computing, Universiti Teknologi Malaysia, Skudai, JB 81310, Malaysia; E-Mail: marazzaque@utm.my; 2 School of Computer Science, University of St Andrews, KY16 9SX, Scotland, UK; E-Mail: simon.dobson@st-andrews.ac.uk

**Keywords:** sensing energy, compressed sensing, adaptive sampling

## Abstract

Sensing of the application environment is the main purpose of a wireless sensor network. Most existing energy management strategies and compression techniques assume that the sensing operation consumes significantly less energy than radio transmission and reception. This assumption does not hold in a number of practical applications. Sensing energy consumption in these applications may be comparable to, or even greater than, that of the radio. In this work, we support this claim by a quantitative analysis of the main operational energy costs of popular sensors, radios and sensor motes. In light of the importance of sensing level energy costs, especially for power hungry sensors, we consider compressed sensing and distributed compressed sensing as potential approaches to provide energy efficient sensing in wireless sensor networks. Numerical experiments investigating the effectiveness of compressed sensing and distributed compressed sensing using real datasets show their potential for efficient utilization of sensing and overall energy costs in wireless sensor networks. It is shown that, for some applications, compressed sensing and distributed compressed sensing can provide greater energy efficiency than transform coding and model-based adaptive sensing in wireless sensor networks.

## 1. Introduction

Wireless sensor networks (WSNs) are critically resource constrained by limited power supply, memory, processing performance and communication bandwidth [1]. Due to their limited power supply, energy consumption is a key issue in the design of protocols and algorithms for WSNs. Energy efficiency is necessary in every level of WSN operations (e.g., sensing, computing, switching, transmission). In the conventional view, energy consumption in WSNs is dominated by radio communications [2–4]. The energy consumption of radio communication mainly depends on the number of bits of data to be transmitted within the network [5]. In most cases, computational energy cost is insignificant compared to communication cost. For instance, the energy cost of transmitting one bit is typically around 500–1,000 times greater than that of a single 32-bit computation [6]. Therefore, using compression to reduce the number of bits to be transmitted has the potential to drastically reduce communication energy costs and increase network lifetime. Thus, researchers have investigated optimal algorithms for the compression of sensed data, communication and sensing in WSNs [4,7].

Most existing data-driven energy management and conservation approaches for WSNs [4,7] target reduction in communications energy at the cost of increased computational energy. In principle, most compression techniques work on reducing the number of bits needed to represent the sensed data, not on the reducing the amount of sensed data; hence, they are unable to utilize sensing energy costs efficiently in WSNs. Importantly, in most cases, these approaches assume that sensing operations consume significantly less energy than radio transmission and reception [7,8]. In fact, the energy cost of sensing is not always insignificant, especially when using power hungry sensors, for example, gas sensors [8–10].

Compressed sensing (CS) provides an alternative to Shannon/Nyquist sampling when the signal under consideration is known to be sparse or compressible [11–13]. Transform-based compression systems reduce the effective dimensionality of an *N*-dimensional signal, *x*, by re-representing it in terms of a sparse or compressible set of coefficients, *α*, in a basis expansion *x* = Ψ*α*, with Ψ an *N* × *N* basis matrix. By sparse, we mean that only *K* ≪ *N* of the coefficients are nonzero and need to be stored or transmitted. By compressible, we mean that the coefficients, *α*, when sorted, decay rapidly enough to zero, so that they can be well-approximated as *K*-sparse. In CS, we measure inner products with *M* ≪ *N* measurement vectors instead of periodic *N* signal samples. In matrix notation, the measurements *y*_*M*_×1__ = Φ*x*, where the rows of the *M* × *N* matrix (Φ) contain the measurement vectors. To recover the signal from the compressive measurements, *y*, reconstruction algorithms search for the sparsest coefficient vector, *α*, that agrees with the measurements [11–13].

CS and DCS (distributed compressed sensing) exploit the information rate within a particular signal. Unlike other compression algorithms, they remove redundancy in the signal during the sampling process, leading to a lower effective sampling rate. Provided certain conditions are satisfied, the signal can still be accurately recovered, even when sampling at a sub-Nyquist rate [11–13]. Even though research on CS and DCS for WSNs is in its early stage, a number of research works, including [14–28], have been published. These works are quite diverse in the issues addressed (e.g., routing, performance, compressive measurements). Like other compression schemes [4,7], most existing CS and DCS works, including [17,20,21,27,29–31], are mainly motivated by the communication cost of WSNs. Very often, these works assume that sensing operations consume significantly less energy than the communications, which may not be true in power hungry sensors, for example, gas sensors [8–10]. Some of these works [16,20–22,24,26,27] have taken care of sensing energy cost implicitly For instance, the authors in [27] consider only the sensing energy cost of the Mica2 motes. Even this cost is not specific to any sensor rather sensor board, including the CPU cost. On the other hand, Charbiwala *et al.* [16] deals with energy-efficient sampling for event detection in WSNs. Similarly, Fazel *et al.* [24] presents random compressive measurements for underwater sensors. Generally speaking, explicit analysis and quantification of sensing level energy efficiency is seldom considered in these works. This could be useful, especially in power hungry sensors in making a trade-off between sensor energy efficiency and QoSissues (e.g., distortion, accuracy) directly related to sensors. Most existing CS/DCS works, including [27,32], compare the performance of CS or DCS or both with other techniques limited to transform coding only. In principle, transform coding does not support sensing-level compression. On the other hand, adaptive sensing-based approaches [33–36] have the potential to minimize sensing level energy cost and improve energy efficiency. Comparison between CS or DCS and model-based adaptive sensing approaches [33–36] could be useful in realizing the potential of CS and DCS. Moreover, most existing works study the energy efficiency or other performances in either periodic monitoring [16,18–22,24–27] or event detection [15,16]. To take a holistic view of the CS and DCS in WSNs, particularly in terms of energy-efficient sensing, consideration of the above issues is important. Therefore, the main objectives of this work are threefold: (i) to quantify sampling or sensing energy cost for a selection of off-the-shelf sensors and to provide a comparative study between operational energy costs of some popular sensor motes when they include these sensors in a WSN; (ii) to show the potential of CS and DCS in providing energy-efficient sensing and other operations (e.g., communication) in WSNs; and (iii) a comparative study between CS and DCS and both model-based adaptive sensing approaches [33–36] and transform coding [7,37] in periodic monitoring and event detection application scenarios.

Section 2 provides a brief overview of related work. Section 3 presents the calculation of operational energy costs in WSNs and a comparative study of popular sensors and sensor motes with respect to these costs. An overview of CS is presented in Section 4. This section also presents CS and DCS in WSNs and their matrices, which will be used in the experimental section. The evaluation in Section 5 presents the results of extensive numerical experiments on CS/DCS in WSNs and shows the potential of these in efficient sensing and overall energy costs. It also includes a comparative study between CS and DCS and their counterparts. Finally, Section 6 concludes the work with some future directions.

## 2. Related Work

Most energy management schemes, especially compression techniques in WSNs, assume that data acquisition or sensing and processing operations consume significantly less energy compared to communication, and so, they work on radio activity minimization [4,7,8]. Authors in [8] have shown that this assumption does not hold in a number of practical applications, where the energy consumption of the sensing operation may be comparable to, or even greater than, that of the communication. In this perspective, they analyzed the power consumptions of some off-the-shelf sensors and radios. Mote-level processing and overall power consumptions are missing in this work, which can work as a useful guide for energy optimization. On the other hand, in [38], the authors calculated the energy cost of various operations, which shows that the sensing energy cost of the sensor is comparable to the cost of the radio. However, this is limited to the XSM (Extreme Scale Mote) platform.

A number of research works have been published on CS and DCS for WSNs. These works are quite diverse in the issues addressed, and compressive measurements and data acquisition is one of the key issues addressed in many of these works (e.g., [21,24,27]). As the main concentration of this work is energy-efficient sensing using CS/DCS, directly related to compressive measurements and data acquisition, this section includes mainly compressive measurements and data acquisition related works.

The authors in [17,31] present DCS-based compressive data gathering (CDG) to compress sensor readings to reduce global data traffic and to distribute energy consumption evenly to optimize network lifetime in large-scale WSNs. The CS-based sparse event detection [15] method shows that the number of active sensors can be greatly reduced, and it can be similar to the number of sparse events, much less than the total number of sources. In [39], the authors optimize the sensing or measurement matrix in DCS. Unlike other joint sparsity models (JSM) [40,41], they exploit different bases for common components and innovations. In particular, they have used the efficient projection (EP) method for optimizing the sensing matrices. In [16], the authors have exploited low power implementation of CS using causal randomized sampling for efficient sampling in event detection. A real-life implementation of the proposed scheme using MicaZ [42] shows the potential of the implementation. The authors in [23] investigate the potential of CS-based signal acquisition for low-complexity energy-efficient ECG(Electrocardiography). compression on a wireless body sensor network mote (Shimmer). They claimed that the implementation of Gaussian random sensing for matrix Φ based on linear transform is too complex, time consuming and is certainly not a real-time task for the MSP430 [43]. To address this problem, they explored three different approaches including binary sparse sensing to the implementation of the random sensing matrix, Φ. The results show that CS is a better alternative to the digital wavelet transform-based ECG compression solutions. In [22,44], a random sampling-based CS has been presented for energy-efficient data acquisition in environmental monitoring using WSNs. The proposed random sampling considers the causality of sampling, hardware limitations and the trade-off between the randomization scheme and computational complexity. Moreover, they used a feedback scheme to adjust the sampling rate of sensors to maintain an acceptable performance. The results show improvements, but no comparison with the deterministic sampling-based CS or other compression techniques. The authors in [24] have proposed random access compressed sensing (RACS) for underwater environment monitoring, which employs random sensing for the sampling and a simple random access for the channel access. RACS improves the network lifetime compared to a traditional network, but a comparison with other compression techniques is missing. Similar to [24], the authors in [21] employ random and non-uniform sampling for compressive measurement by exploiting heterogeneity in WSNs and exploit spatial correlation to find the compressive measurements. The results show that non-uniform sampling outperforms uniform (Bernoulli) sampling. The authors in [20] have exploited CS to improve network lifetime. Like most existing works on CS and DCS for WSNs, this work has not considered the sensing cost in calculating network lifetime. Even they disregarded the listening and computational costs in their calculations, but these costs, especially the listening one, are not always insignificant [45,46].

In [47], the authors have presented compressive distributed sensing using random walk, an algorithm for compressive sensing in WSNs using rateless coding. The algorithm is independent of routing algorithms or network topologies and delivers the benefit of using non-uniform and unequal error protection codes. In [25], a mixed algorithm by integrating pack and forward and DCS is presented to minimize the number of packets to transmit in WSNs. Results show that the algorithm has the potential to prolong the lifetime of the network, achieving a trade-off between traffic in the network and energy. The work in [26] has shown how to select the measurement matrix and a representation basis for a specific application of CS. Through extensive numerical experiments, it has shown that both uniform and random samplings outperform Gaussian sampling. It has also claimed that Gaussian sampling or scheduling is not practical for soil moisture monitoring, and that could be true in some other applications, as well. In [28], the authors have addressed the efficient compressive sampling of spatially sparse signals in sensor networks. In particular, they have introduced an atypical CS sampling scheme for spatially sparse bi-dimensional signals. Analytical results show the potential of the scheme. Real implementation or real sensor node-based analysis is missing. In a very recent work [27], the authors have analyzed the energy efficiency of CS in WSNs. Unlike most existing works in this area, this paper includes the sensing cost in their modeling and experiments. Numerical analysis-based results show that CS has the potential to improve network lifetime in WSNs compared to transform coding and no processing scheme. This work complements our work, but a few issues are missing, which could be useful in a number of WSN applications. It has considered sensing cost integrated with processing cost, and the results are limited to the Mica2 [48] mote only. Moreover, it has considered Gaussian and deterministic sampling, which may not be practical in some WSN applications, and a comparison with other compression approaches, particularly mode-based active or adaptive sensing approaches [34–36,49], which have sensing-level compressibility, is missing.

In summary, existing works, including [17,29–31], exploit CS or DCS at the gathering level, assuming that all sensors sample the physical phenomenon at each sampling instance. Thus, they are missing the acquisition- or sensor-level compression, which is one of the key benefits of CS and DCS. On the other hand, works, including [21,23,24,26,27,44,50,51], have applied CS/DCS at the sensing level, but explicit consideration of sensing energy cost analysis and efficiency is disregarded in these works. Very few [27] of them considered, but detail, the calculation of sensing energy cost using the sensors' information, e.g., start-up time, response time, *etc.*, which could play an important role in the sampling rate, and sensor-related QoS (e.g., accuracy) is missing. Similar to CS and DCS, model-based active or adaptive sensing [4,7,34–36,49] could integrate the signal acquisition and compression steps into a single process. Therefore, comparison between model-based active or adaptive sensing and CS/DCS, which is missing in most existing works, could be useful. Finally, most existing works study the energy efficiency of CS/DCS or other performances in periodic monitoring or event detection applications, not both.

## 3. Operational Energy Costs in WSNs

In WSN applications, the energy used by a node consists of the energy consumed by computing, receiving, transmitting, listening for messages on the radio channel, sampling data and sleeping. The switching of state, especially in the radio, can also cause significant energy consumption. In the following, we briefly discuss these operational energy costs in WSNs and focus on energy consumption during a single sampling period. In the calculation of these operational energy costs in a sensor node, we consider the MACprotocol, as it has a significant impact on energy consumption. Here, we consider the popular BMAC (Berkeley Media Access Control) [45]. Its parameters (for details, please see [45]) related to the energy costs calculation are summarized as: sampling period (*S*) = 360 s, neighborhood size (*n_b_*) = 5, channel check interval (*CCI*) = 0.1 s, check time (*T_ch_*) = 0.000128 and preamble (bytes) = 3,144. For simplicity, we consider a common sampling period of 360 s for all sensors. Even though this period may cause under-sampling for some sensors, considering the high startup and response time of some sensors, we need to have this low sampling rate. Justification for the selection of other values is available in [45,46]. In calculating energy consumption, we use the maximum values of parameters for the worst case analysis.

### 3.1. Sensing Energy Cost

Due to the wide diversity of sensors, the power consumption of sensors varies greatly. For passive sensors, such as passive light or temperature sensors, power consumption is negligible in comparison to other devices on a wireless sensor node. On the other hand, for active sensors, such as sonar, soil and gas sensors, power consumption can be significant [8]. Each sensor node can include several sensors, and each of these sensors typically has its own energy consumption characteristics and, in some cases, its own sampling frequency. In general, a sensor, *i*, will have the following sensing energy consumption.

(1) $$E_{sm}=V_{dc}*I_{i}*T_{i}$$

where *T_i_* is the time required for obtaining a single sample from sensor *i* and *I_i_* is the current draw of sensor *i*. *T_i_* depends on the start-up (*T_s_*), response (*T_r_*) and measurement (*T_m_*) times of the sensors. As *T_m_* is small in comparison to *T_s_* and *T_r_* for most sensors, we consider only *T_s_* and *T_r_* in calculating *T_i_*.

The startup time (*T_s_*) is the time required for a sensor to reach the ready state after power is engaged, upon which the sensor can give the correct value. It is a well-known factor in the power management of sensors [59]. If a sensing task does not wait for the *T_s_* after the micro controller unit (MCU) requests the sensor to turn on, the task will receive the wrong value. *T_s_* varies significantly between sensor types. As shown in Table 1, a temperature sensor (SHT1X [56]) needs only 0.011 s to become ready, whereas both VOC (Volatile Organic Compound) [10] and CO_2_ (Carbon Dioxide) [9] sensors require more than 3 min. Sensors do not change output state immediately when an input parameter or environmental parameter change occurs. Rather, they change to the new state over a period of time, called the response time (*T_r_*). *T_r_* can be denned as the time required for a sensor output to change from its previous value to a final settled value within a tolerance band of the correct new value [60]. Response time depends on the sensor type, its working principle and the environment in which it is used. Due to space limitations, we report on a few popular sensor types. Table 1 presents the list of sensors investigated and their *T_s_*, *T_r_* and *E_sm_* values. From Table 1, it is very evident that the power or energy requirements of the sensors are very diverse (e.g., 0.0048 mJ to 225,000 mJ).

### 3.2. Computational Energy Cost

The computational energy cost (*E_comp_*) of sensor motes is a key constituent of the overall operational energy costs in WSNs. *E_comp_* includes the MCU's active mode and other modes' (e.g., standby/idle/sleep) energy consumption. It is often disregarded, as it is insignificant compared to communication energy, but in cases of complex mathematical operations (floating point, matrix multiplication) or very long sleep times for the MCU (e.g., during sensor startup time, response time), it can be significant. *E_comp_* can be expressed as:

(2) $$E_{\text{comp}}=V_{dc}*I_{\text{mcu}–\text{active}}*T_{\text{mcu}–\text{active}}+V_{dc}*I_{\text{mcu}–\text{sleep}}*T_{\text{mcu}–\text{sleep}}$$

where *I_mcu_*_–_*_active_* and *I_mcu_*_–_*_sleep_* are the MCU active and sleep mode current, respectively. *T_mcu_*_–_*_active_* and *T_mcu_*_–_*_sleep_* are the MCU active and sleep modes durations, respectively.

### 3.3. Communication Energy Cost

The communication energy cost, *E_comm_*, is conventionally the most important constituent of the operational costs in WSNs. The constituents of *E_comm_* are listening, transmission, reception, sleeping and switching energy.

The transmission energy, *E_tx_*, component of *E_comm_* refers to the energy consumed during the transmission of packets. *E_tx_* can be expressed as:

(3) $$E_{tx}=V_{dc}*I_{tx}*P_{b}*T_{b}$$

where *I_tx_* is the current consumption in the transmission mode of the radio. *P_b_* is the bit length of the packet to be transmitted along with the preamble for BMAC (e.g., based on BMAC packet format for a two-byte payload, *P_b_* = *L_preamble_* + *L_packet_* = (3125 * 8 + 19 * 8)*bits* [45]), and *T_b_* is the transmission time of a single bit.

The reception energy, *E_rx_*, component of *E_comm_* refers to the energy consumed when receiving packets. *E_rx_* can be expressed as:

(4) $$E_{rx}=V_{dc}*I_{rx}*T_{br}*T_{b}$$

where *I_rx_* is the current consumption in reception mode and *P_br_* is the bit length of the packet to be received along with the preamble for BMAC, which can vary from *P_b_* to *n_b_P_b_*. Therefore, a node can receive more than one packet during one sampling period.

The listening energy, *E_listen_*, is the radio energy consumption when the radio is active, but not receiving or sending packets. This listening is to check for messages on the radio channel [45], and it, if possible, should be duty cycles, *i.e.*, low power listening. *E_listen_* can be expressed as:

(5) $$E_{\text{listen}}=V_{dc}*I_{\text{listen}}*T_{\text{listen}}$$

where *I_listen_* is the current draw of the radio in listen mode and *T_listen_* is the time in each sampling period that the radio stays in listen mode, which depends on the MAC protocol. For BMAC [45,46] $T_{\text{listen}}=\frac{S}{\text{CCI}}*T_{ch}$, where *S* is the sampling period, *CCI* is the channel check interval and *T_ch_* is the time during which the node is awake in every *CCI*, and values used for these variables were presented earlier. For popular radios, like CC2420, CC1000 [61,62], *I_listen_* can be approximated by *I_rx_*, or the receive mode current [46].

Switching states in the radio and MCU are regular occurrences in WSNs. Switching cost *E_sw_* for the MCU is not significant. However, the cost of switching the radio [46] is not negligible. For the radio, the following equation determines the energy consumed for the switching state:

(6) $$E_{sw}=\frac{\left|\left(I_{st_{j}}-I_{st_{i}}\right)\right|*T_{st_{ij}}*V_{dc}}{2}$$

where *I_st_j__* is the current draw of the radio in the state switched to, and *I_st_i__* is the current draw of the radio in the current state and *T_st_ij__* is the time required for the radio to go from state *i* to *j*. If a radio switches from sleep mode to transmission or receive mode, it uses wake-up-time as *T_st_ij__*; otherwise, it is the switching-time.

The sleep time, *T_slp_*, is simply the time remaining that is not consumed by other operations.

(7) $$E_{\text{slp}}=V_{dc}*I_{\text{slp}}*T_{\text{slp}}$$

where *I_slp_* is the current draw of the radio in sleep mode, and *T_slp_* = *S* − (P_b_*T_b_ + *P_b_r* *T_b_ + *T_listen_* + *T_d_* + *T_st_ij__*/2), where *T_d_* = *T_i_* + *T_mcu_*_–_*_active_*.

Using Equations (3)–(7), we have calculated (see Table 2) the overall communication energy costs of a few popular radios. Table 2 clearly shows that for BMAC, the energy cost of switching is the main contributor of *E_comm_*. This is because the radio needs to switch between sleep and listening mode (*S*/*CCI* = 360/0.1 = 300) 3,600 times during the sampling period (360 s). On the contrary, in IEEE 802.15.4 MAC, *E_comm_* is dominated by the *E_l_*, as its node needs to be awake for long periods of time (it could as high as 54 time slots, which is 17 ms in TelosB) [46].

### 3.4. Comparison of *E_sm_*, *E_comp_* and *E_comm_*

We present a comparison of *E_sm_*, *E_comp_* and *E_comm_* for three popular sensor motes [48,65,66], where they include the sensors listed in Table 1. Comparisons are normalized with respect to communication energy *E_comm_*. Table 3 presents the normalized energy consumptions (approximated). It is obvious that sampling energy is not always insignificant, especially in the case of power hungry sensors, such as gas, flow control, level sensor, *etc.* For instance, in the case of the accelerometer MMA7260Q [52], *E_sm_* is only 0.0000268 times *E_camm_* (in TelosB/Imote2), but it becomes 1,249.25 times *E_comm_* in the CO_2_ sensor, GE/Telaire 6004 [9]. Almost a similar trend follows if we compare *E_comp_* and *E_sm_* in the case of TelosB and Mica2. Along with higher voltage and current requirements, longer startup (e.g., preheating in CO_2_ or VOC sensors) and response time are mainly responsible for these higher values of *E_sm_*. Sensors with longer *T_s_* and *T_r_* have higher *E_comp_*, as they keep the MCU in active mode for longer times; hence, their energy consumption is greater. As shown in Table 3, in the case of the accelerometer, MMA7260Q [52] (in TelosB), *E_comp_* is only 0.044 times *E_comm_*, but it becomes 9.03 times *E_comm_* for the CO_2_ sensor GE/Telaire 6004 [9], as it has longer *T_s_* and *T_r_*. For the Imote2, due to the high current consumption of the MCU in active mode, sensors with lower *T_s_* and *T_r_* have relatively higher *E_comp_* compared to the sensors with higher *T_s_* and *T_r_*. For sensors with lower *T_s_* and *T_r_*, *E_sm_* is insignificant compared to *E_comp_*, and in the case of sensors with higher *T_s_* and *T_r_*, it is comparable to *E_comp_* in most cases. As TelosB and Imote2 use the same CC2420 radio, for the same the sensor, they have the same *E_sm_* value.

## 4. Compressed Sensing

The CS field has existed for at least four decades, but recently, researchers' interest in the field has exploded, especially in the areas of applied mathematics, computer science and electrical engineering, due to several important results obtained by Donoho, Candes, Romberg and Tao [11,67,68]. CS is a novel sensing paradigm that goes against the traditional understanding of data acquisition and can surpass the traditional limits of sampling theory. It is also known as sub-Nyquist sampling, and it has a surprising property that one can recover sparse signals from far fewer samples than is predicted by the Nyquist-Shannon sampling theorem [11–13]. On the other hand, down sampling methods (e.g., [35,36]) cannot work with reasonable accuracy using a sampling rate less than the Nyquist rate. CS/DCS works at a sub-Nyquist rate (M is considered to be always less than the Nyquist rate); still, it can be recovered with high accuracy if certain conditions (e.g., sparsity and incoherence) are satisfied [11–13].

The notion of CS/DCS [12,67] exploits the fact that there is a difference between the rate of change of a conventional signal and the rate of information in the signal. CS/DCS exploits the information rate within a particular signal. Redundancy in the signal is removed during the sampling process itself, leading to a lower effective sampling rate and lower energy consumption (*E_sm_*). The signal, sampled at this lower (sub-Nyquist) rate, still can be recovered with high accuracy [12,69].

### 4.1. Overview of Compressed Sensing

The earlier part of this section briefly summarizes the key elements of CS/DCS that are required in the later part of this section. For more advanced and detailed information on CS theory, readers are referred to [11–13] and the references therein.

### 4.1.1. Signal Representation

One of the preconditions for any signal to be compressible by means of CS/DCS is that the signal is sparse or compressible. Consider *x* to be a discrete signal given by the vector, *x*, of size *N*. Given a basis, ${\left\{\psi_{i}\right\}}_{i=1}^{N}$ we can represent every signal *x* ∈ ℝ*^N^* in terms of coefficients ${\left\{\alpha_{i}\right\}}_{i=1}^{N}$ as $x={\text{∑}}_{i=1}^{N}\alpha_{i}\psi_{i}$; putting the *ψ_i_* as columns into the *N* × *N* matrix, Ψ, we can represent *x* compactly as *x* = Ψ*α*. This matrix, Ψ, may be referred to as the representation matrix or basis. A signal, *x*, is *K*-sparse if ‖x‖_0_ ≤ *K*, which means only *K* ≪ *N* entries are nonzero. Many natural and man-made signals are not strictly sparse, but can be approximated as such. These are known as compressible signals.

### 4.1.2. Compressive Measurement

CS integrates the signal sampling and compression steps into a single process [11–13]. In CS, we do not acquire *x*, but rather, acquire *y_M_*_×1_ = Φ*x* linear measurements or samples using an *M*×*N* measurement matrix, where *M* ≪ *N*. This linear measurement, also known as a projection of *x* onto *M*, compressively samples *y* according to a projection matrix, Φ [11,70]. In order to have higher signal “compression” during sampling, we need to make *M* as close as possible to *K*. The matrix, Φ, represents a dimensionality reduction, as it maps ℝ*^N^* into ℝ*^M^*, where *M* ≪ *N*. Usually, in a standard CS framework, the measurements are non-adaptive. In certain settings, adaptive measurement schemes can be useful [13]. In order to recover a good estimate of *x* from the *M* compressive measurements, the measurement matrix, Φ, should satisfy the restricted isometry property (RIP) [26,71].

### 4.1.3. Reconstruction Algorithm

The reconstruction problem of the original signal, *x*, expressed by *x* = Ψ*α*, is to determine *α* for a given measurement *y* = ΦΨ*α* and known matrices Φ and Ψ. This is an under-determined linear system, as the number of equations, *M*, is much smaller than the number of variables, *N* (*i.e.*, the number of entries of *α*). Hence, there are infinitely many signal coefficient vectors, *x′*, that produce the same set of compressive measurements *y* = Φ*x*, and to recover the “right” signal, we need to exploit *a priori* knowledge of its sparsity or compressibility.

In practice, stable recovery algorithms rely on the RIP, hence requiring at least *M* = *K*log(*N*/*M*) measurements. These recovery algorithms can be grouped into three types: (i) *l*_1_ minimization; (ii) greedy approach; and (iii) combinatorial approach [13]. A number of algorithms fall into the *l*_1_ minimization category [11,13,68,72–74]. Algorithms, such as matching pursuit [75], orthogonal matching pursuit [76], StOMP [77], *etc.*, are examples of the greedy approach, and the algorithm presented in [78] is an example of the combinatorial approach.

### 4.2. CS in WSNs

Considering the inherent inefficiencies of transform coding and the availability of sparsity or compressibility in WSNs signals due to spatio-temporal correlations within the sensor readings, CS and DCS are gaining researchers' attention as potential compression approaches for WSNs (e.g., [14,15,31,79,80]). The asymmetric computational nature of CS and DCS makes them even more attractive for compression in WSNs. In CS and DCS, most computation takes place at the decoder (sink), rather than at the encoder (sensor nodes); thus, sensor nodes with minimal computational performance can efficiently encode data. In addition, CS has two further advantages: graceful degradation in the event of abnormal sensor readings and low sensitivity to packet loss. Hence, CS and DCS are promising approaches [81,82] for removing redundancy during sensing operations in WSNs,and, hence, for energy efficient sensing.

CS for WSNs exploits only temporal (intra-signal) structures within multiple sensor readings at a single sensor and does not exploit spatial (inter-signal) correlations amongst nearby sensors [26]. DCS works on multi-sensor scenarios considering only standard CS for the joint measurements at single time instances (e.g., [29]). These schemes ignore the intra-signal or temporal correlations. On the other hand, some DCS approaches (spatio-temporal) [83,84] exploit the spatial correlation structures between nearby sensors and the temporal correlation of each sensor's time variant readings.

### 4.3. Signal Measurement and Representation in WSNs

In this section, we briefly present the selection of a measurement matrix, Φ, and a representation basis, Ψ. The measurement matrix, Φ, directly corresponds to the measurement or sampling scheduling of a WSN application, whereas the representation basis, Ψ, is used in signal sparsifying and reconstruction algorithm to determine *α* and then recover the original signal, *x*.

### 4.3.1. Measurement or Projection Matrix Φ

The measurement or projection matrix mainly depends on the signal of interest, whose detail may be unknown to a user. It is highly unlikely that the user will know ahead of time which *K* coefficients give the best *K*-term approximation (*i.e.*, the ordering *α*(1),…, will not be known), and the measurement or projection matrix, Φ, may not be known either. There are two possible solutions to this problem: (i) machine learning; and (ii) random projection. Learning the properties of the signal of interest and then generating a measurement or projection matrix in WSNs can be expensive in terms of computation and communication cost. Work [11,70] on compressed sensing has shown that random projections can guarantee the recovery of a near-optimal approximation of compressible data, with very little degradation of performance. In the order of *O*(*K* log(*N*)), random projections of the data can produce an approximation with error comparable to the best approximation error using the *K*-largest transform coefficients [85]. A number of existing CS and DCS works exploit an independent and identically distributed (i.i.d.) Gaussian or Bernoulli/Rademacher (random ±1) matrix for random projection, as they provide a very useful universal measurement or projection basis, which is incoherent with any given representation basis, Ψ, with high probability. Existing works, including [17,18,20,27,29,31,79,86], use one of these matrices to generate Φ. On the contrary, existing works, including [21,23,24,26,44,51], claim that these matrices are not suitable in a number of WSN applications, as they are dense, virtually non-zero-entries. Computing a single random projection of the sensor data via such dense measurements would require sensing and accessing the values at all the sensor nodes [87]. This clearly defeats the basic objective of CS, minimizing the amount of measurements taken. Moreover, the computation of such a projection is too complex, time consuming and may not be a real-time task for low power microcontrollers [23]. Therefore, sparse random measurement matrices are necessary, especially for energy-efficient sensing, and these have been considered in [21,23,24,26,44,51]. Moreover, sparse random projections can reduce computational complexity, minimize communication cost and even be exploited to reduce decoding complexity [51].

In WSNs, sensors can obtain a Φ from the sink (centralized) [17,31], or they can generate it using the same pseudo-random number generator at all nodes, including the sink [21,24,26,44]. Once sensor nodes in WSNs know Φ, they can calculate the compressive measurements by projections of the data, *x*, onto the measurement vectors, *y_i_* =< Φ*_i_*, *x* >; Φ*_i_* is an *i^th^* row of Φ. In the case of temporally correlated signals, it is easy to find the compressive measurements, as it is within a sensor node, but in the case of spatially correlated signals, distributed computation and communication amongst neighboring nodes adds complexity. Routing plays an important role in DCS [14], especially in the case of dense random projections. On the other hand, if the measurement matrix does not change through the lifetime of the WSNs, the sensor nodes can be preloaded with this data before deployment [27].

### 4.3.2. Representation Basis Ψ

Representation basis in CS or DCS depends on the nature of the signal of interest. There are two main criteria in selecting a good representation basis (Ψ): (i) its corresponding inverse has to sufficiently sparsify the signal, *x*; and (ii) it has to be sufficiently incoherent with the corresponding measurement matrix, Φ. Finding such a basis is not a trivial job, considering the sparseness of the measurement matrix, Φ. We can find a basis that satisfies the above two criteria without assuming *a priori* knowledge of the signal, except its size (which determines the size of the matrix). However, this can be time consuming, as it may take a large number of trial-and-error steps to find the basis. Hence, typically certain known features of the signal are taken into account in searching for a suitable basis to speed up this design process [21,26,71]. Based on the nature of WSNs application signals (temporal and spatial), we can use the Fourier transform (FT), discrete cosine transform (DCT), wavelet transform (Haar, Daubechies), *etc.* [37], bases for sparse representation of the signals. Typically, the DCT is suitable for smooth signals, whereas wavelet-based transforms are more suitable for piecewise constant data [26,88]. A combination of more than one of these transforms can be exploited for better sparse representation of the signals [89,90].

### 4.4. CS/DCS in Sensing and Overall Energy Efficiency

Calculation of sensing energy efficiency or savings is necessary in studying the potential of CS/DCS as an energy-efficient sensing method in WSNs. In calculating the sensing energy efficiency and the overall energy efficiency due to CS/DCS, we need to define the sampling ratio (SR) (compression ratio in CS/DCS). This is the ratio of the number of samples collected when compression is not used, *s_r_*, to the number of samples collected when compression is used, *s_c_*, and is given by:

(8) $$SR=\frac{s_{r}}{s_{c}}$$

The percentage saving in samples is given by $\left(1-\frac{1}{SR}\right)\times 100\%$. For most compression algorithms, *SR* = 1. However, CS/DCS allows *SR* > 1. In CS/DCS, a temporally or spatially correlated signal of length *N* with *K*-sparse representation only *M* = *O*(*K* log *N*) incoherent measurements rather than *N* samples is sufficient to recover the signal with high probability, where *K* ≪ *N*. Therefore, SR can be expressed as:

(9) $$SR_{cs}=\frac{N}{M}$$

Sensing energy saving merely depends on the measurement matrix, Φ; precisely how it is obtained. As we mentioned earlier in the measurement matrix section, making measurements in CS/DCS using sparse random measurement matrices is preferable for energy-efficient sensing compared to a dense measurement matrix as a linear combination of all the measurements. Hence, similar to [21,23,24,26,44,51], this work will consider this thusly. Moreover, considering the complexity, this work will consider the pseudo-random matrix, as mentioned earlier. This work also assumes that this pseudo-random generation maintains the causality of the sampling process [44].

In CS implementation, at every sampling period, a sensor node tosses a coin to determine whether it participates in sensing (with probability $p=\frac{M}{N}$, where *N* is the total number of temporally correlated samples in non-compression mode) or stays inactive (with probability 1 − *p*) during that period. If it participates, it measures the physical quantity of interest and encodes and sends it to the base station. In the case of DCS (for spatially correlated signals), at the beginning of a frame (after sensing if all the selected sensors start sending at the same time, collision is unavoidable, so multiple-access schemes, like TDMA, CSMA/CD, *etc.*, and their frame concept are needed), each sensor node tosses a coin to determine whether it participates in sensing (with probability $p=\frac{M}{N}$, where *N* is the total number of nodes in the network or cluster) or stays inactive (with probability 1 − *p*) during that frame. If a node is selected for sensing, it measures the physical quantity of interest, encodes it into a packet and sends it to the base station. Thus, a subset, *M*, of *N* sensors is selected at random to conduct measurements. Randomly selecting a subset of the total number of sensors in a WSN, one can perform the compression directly in the spatial domain [21,24,26,44]. Based on the above discussion and using Equation (9), we can approximate the sensing energy saving (*E_sm_savings__*) in CS/DCS using sparse and pseudo-random measurement by means of the following equation.

(10) $$E_{sm_{\text{saving}}}\approx \left(\frac{N-M}{N}\right)(E_{sm})$$

According to the theory of CS, [12,68,91] states that as long as the number of observations, *M*, picked uniformly at random, is greater than *KClog*(*N*), then, with very high probability, the reconstruction will be perfect. Here, *C* is a constant that is independent of *N* and *K*. In particular, as suggested by the “four-to-one” practical rule introduced in [12], *M* = 4*K* is generally sufficient for exact recovery, which means one needs about four incoherent samples per unknown nonzero term. In case of DCS, to find exactly *M* sampling nodes out of *N* available nodes in a network or cluster, a good amount of coordination is needed by the nodes. The use of random sampling-based probabilistic methods do not require exactly *M* sampling nodes, but, rather, require the mean number of sampling nodes to be *M*. These methods require less coordination among the nodes and are more suited for DCS [21,24].

Like other data-driven energy management and conservation approaches for WSNs [4,7], most existing CS/DCS works on WSNs target the reduction in communications energy at the cost of increased computational energy. Energy savings in communication *E_comm_* and computation *E_comp_* depend on the implementation of CS/DCS. If CS is implemented in a single node, then temporal correlation can be exploited and, then, *N* – *M* communications can be saved (considering every sample is communicated to the base station separately using a single hop, if multi-hops are used, then this needs to multiply with the hop counts) compared to the baseline or classical non-compression-based *N* communications. Applying this approach at the multi-node level, *N*^2^ – *MN* communications can be saved compared to the baseline *N*^2^ communications [17,29,31]. On the other hand, in case of spatially correlated signals, if only *M* sensors out of *N* sensors send their readings, then *N* – *M* communications can be saved compared to the baseline *N* communications (considering every sample is communicated to the base station separately using a single hop, if multi-hops are used, then this needs to multiply with the hop counts.). Thus reduced number of sensor readings also reduces the *E_comm_* and *E_comp_*, as using CS, a sensor needs to process and send fewer readings. In the case of DCS, fewer numbers of sensor nodes sense, process and send their readings. In both cases, if only *M* required samples are collected instead of *N*, then savings compared to the no-compression situation in *E_comm_* and *E_comp_* are proportional to the factor (considering every sample is communicated to the base station separately using a single hop, if multi-hops are used, then this needs to multiply with the hop counts.), $\frac{N-M}{N}$. These savings come at the cost of additional encoding or computational cost *E_encoding_cs__* in obtaining the measurement matrix and reconstruction error (*E_r_*). In the case of a dense random projection matrix (e.g., [17,31]), *E_encoding_cs__* could be very high, but in a sparse and pseudo-random matrix it can be minimized significantly [21,24]. Moreover, in the DCS implementation, it could be high due to pre-processing communication amongst the nodes. Therefore, the overall energy cost savings in CS/DCS using sparse and pseudo-random measurements can be approximated as below:

(11) $$E_{\text{saving}}\approx \left(\frac{N-M}{N}\right)(E_{sm}+E_{\text{comm}}+E_{\text{comp}})-E_{\text{encoding}g_{cs}}(M)$$

Like any other compression technique, in CS/DCS, measurement of the accuracy of the reconstruction algorithm is important. One popular way to do this is by calculating the root mean-squared error (RMSE) values normalized with respect to the *l*_2_ norm of the signal [21,26,92]. This can be expressed as below:

(12) RMSE=‖x−x^‖2‖x‖2

where *x̂* is the approximated signal and ${\left\|x\right\|}_{2}=\sqrt[{2}]{{\text{∑}}_{i=1}^{n}x_{i}}$ denotes the 2-norm of *x*.

## 5. Evaluation

This section evaluates the effectiveness of CS/DCS as an energy-efficient sensing in WSNs using the algorithms introduced in the previous section. It also includes the overall energy savings of CS/DCS in WSNs. For the evaluation, a numerical experiment has been used. Two comparative studies have been conducted for two different types of signals to show the potential of CS/DCS in comparison to its counterparts, including the down sampling method [35,36]. For temporally correlated signals, a comparison was made between CS, transform coding (TC) and adaptive sampling-based predictive coding (PC). For spatially correlated signals, it was between DCS, TC and ASAP (adaptive sampling approach) [36]. Adaptive sampling-based predictive coding (PC) and ASAP are the two down sampling methods in the study. DCS implementation has considered a clustered WSN and assumed that clusters are formed based on the spatial correlation.

For the evaluation, we used three real-life sensor datasets with different sampling rates (e.g., very low, low [93]). Dataset one is from the Intel Lab Data [94], the second one from the Harvard's volcanic eruption monitoring project [95] and the final one from the BeACON project [96]. The first dataset is for temperature, the second one for seismic waves and the final one for CO_2_ emissions. In dataset one, data was collected from 54 sensors deployed in the Intel Berkeley Research lab between February 28th and April 5th, 2004 [94]. Mica2Dot [97] motes with weather sensor boards collected time-stamped topology information, along with humidity, temperature, light and voltage values at a sample rate of 1/31. The second dataset is from the raw seismic signals collected during the August, 2005, Reventador Volcano, Ecuador, deployment. This project [95] used TMoteSky sensor [98] nodes and a sampling rate of 100 to collect these readings. The third and final dataset is taken from the BeACONproject's Skyline High School site for the month of August, 2012 [96], which sampled C0_2_ readings once every 5 min. The BeACON project hardware was more powerful than typical WSN nodes, and the nodes were connected to main power. Hence, for analysis in a WSN environment, we assumed the hardware to be similar to the CitySee project [99], that is, TelosB [65] nodes and GE/Telaire 6004 [9] CO_2_ sensors.

Due to the unavailability of the implementation detail of the BeACON project and the spatial information of sensor nodes in the project [95], we considered these datasets for temporal correlation only; hence, only CS was applied. Additionally, dataset one was considered for temporal and spatial correlation; hence, CS and DCS were applied. To perform the experiments, we divided each dataset into windows of *N* samples. Even though these applications can tolerate some delay, the inclusion of too many samples could cause unacceptable delay, especially for CO_2_, as their sampling frequency is quite low compared to temperature. Hence, *N* = 512 for CO_2_, and *N* = 1,024 for temperature and volcanic data. Dividing the dataset into windows of *N* samples allows us to balance the computational complexity/delay and estimation accuracy. For real-time or close-to-real time applications, it is desirable to use smaller *N*. On the other hand, larger *N* generally results in better estimates, provided that the data statistics are stationary, at the cost of increased computational complexity [26].

For the evaluation, we used Matlab and the Sparse Lab [100]. As our main objective is to study the potential of CS/DCS in energy-efficient sensing for WSNs, rather than assessing the performance of the reconstruction algorithms of CS/DCS, we use a standard reconstruction algorithm (Basis Pursuit [72]). Haar wavelet transform was used for sparsification. As the Haar wavelet basis requires *N* to be a power of two (dyadic), we consider *N* = *2^p^*, where *p* = 9,10,11 for temporal data. In the case of spatial data for DCS, *p* = 5 and 6, means 32 and 64 nodes are needed, respectively. The Intel dataset [94] has only 54 nodes, so we have added 10 more nodes with their approximated readings. Approximated readings for the added sensors were based on nearby sensors' spatial correlation statistics [101].

The results are presented in three parts. The first part presents the sparsity of the datasets used and the potential of CS/DCS as energy-efficient sampling in WSNs. The second part quantifies the amount of savings, due to CS/DCS in sensing, and the overall energy costs of WSNs. The third and final part presents the comparative study. In all three parts, we used sensing energy cost savings, overall energy cost savings, absolute mean reconstruction error (*R_mean_*) and root mean-squared error (RMSE) as performance analysis parameters. Typical WSN applications fall into two categories: periodic monitoring and event detection. Hence, in the experiments, we did the analysis for both. For the results calculation, we ran each experiment 100 times and calculated the average. Every figure of the evaluation section contains two parts: (a) showing signal reconstructions; and (b) showing residual errors for the corresponding reconstructions.

### 5.1. Sparsity Analysis and the Potential of CS/DCS in WSNs

**Figure 1.**
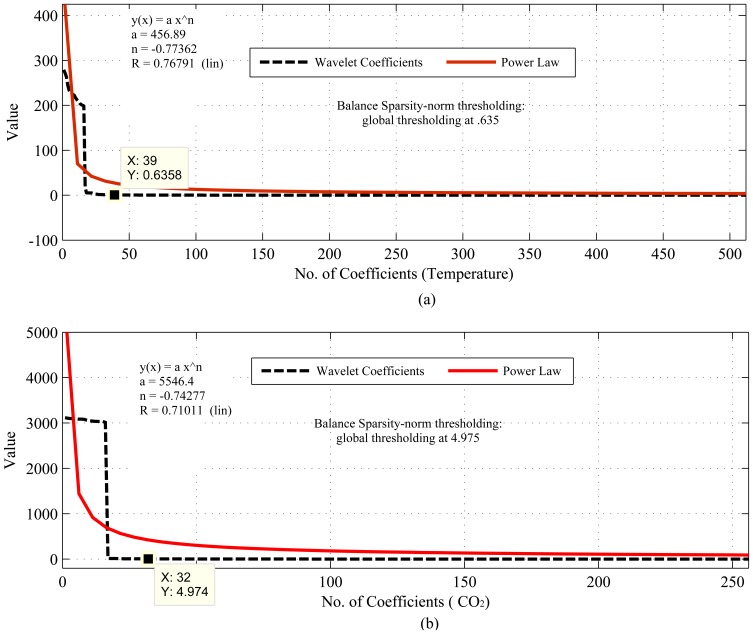
Sparsity analysis of temporally correlated temperature and CO_2_ emission readings [96] using discrete wavelet transform (DWT).

**Figure 2.**
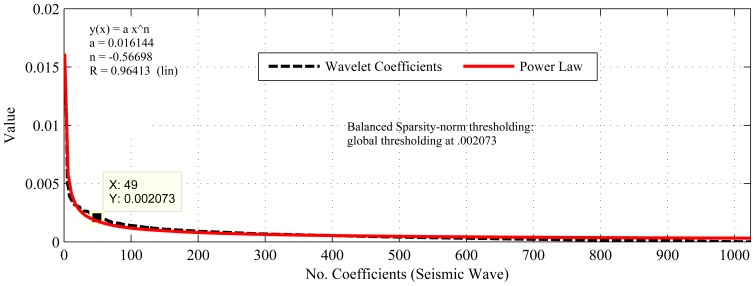
Sparsity analysis of a temporally correlated seismic signal [95] using DWT.

**Figure 3.**
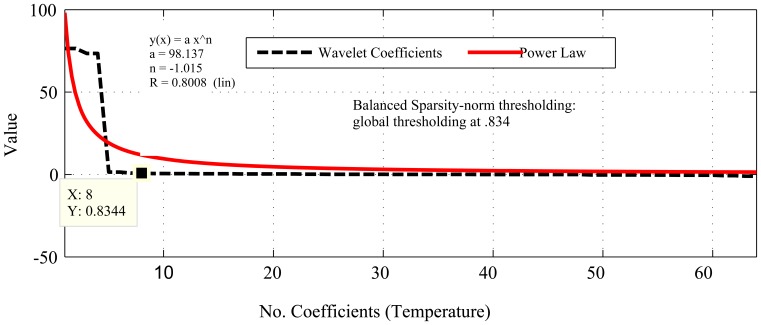
Sparsity analysis of spatially correlated temperature data [94] using DWT.

**Figure 4.**
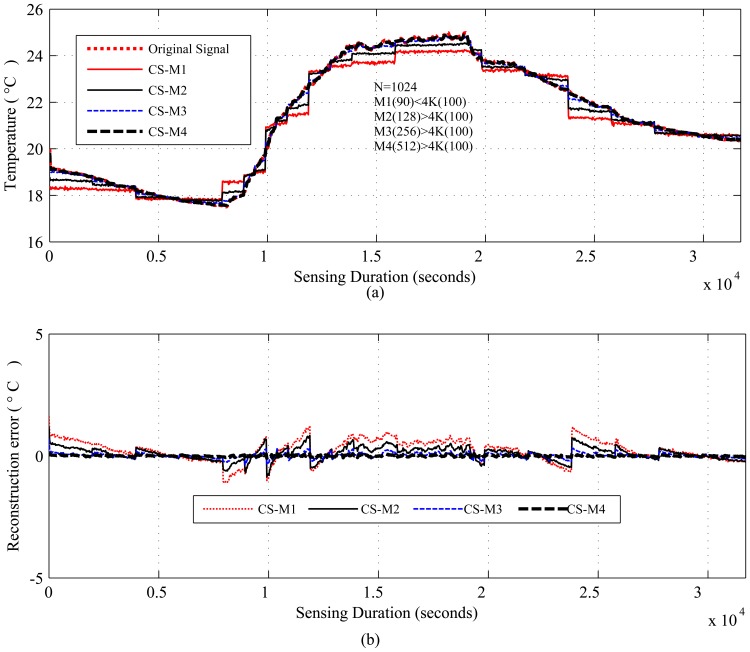
Compressed sensing (CS) in temporally correlated temperature [94] signals.

**Figure 5.**
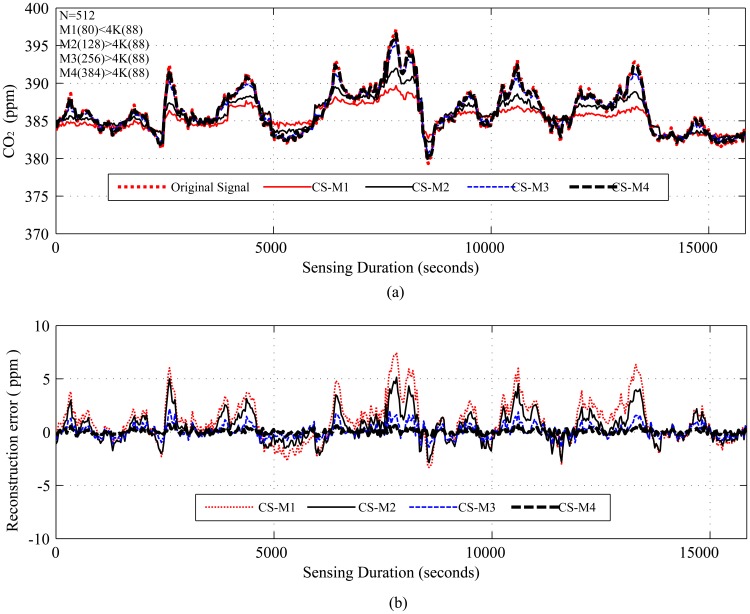
CS in temporally correlated CO_2_ readings [96].

**Figure 6.**
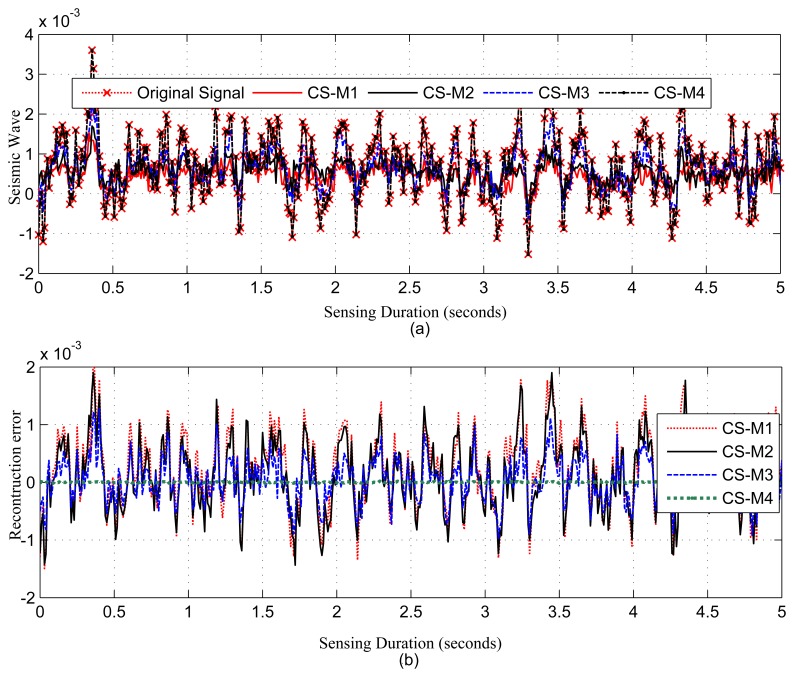
CS in a temporally correlated seismic wave.

**Figure 7.**
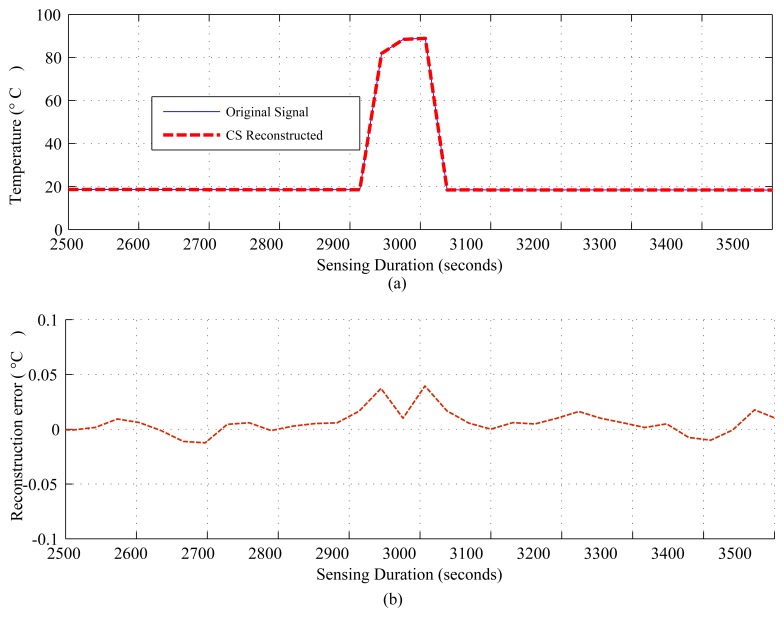
CS-based event detection in a temporally correlated temperature [94] signal.

**Figure 8.**
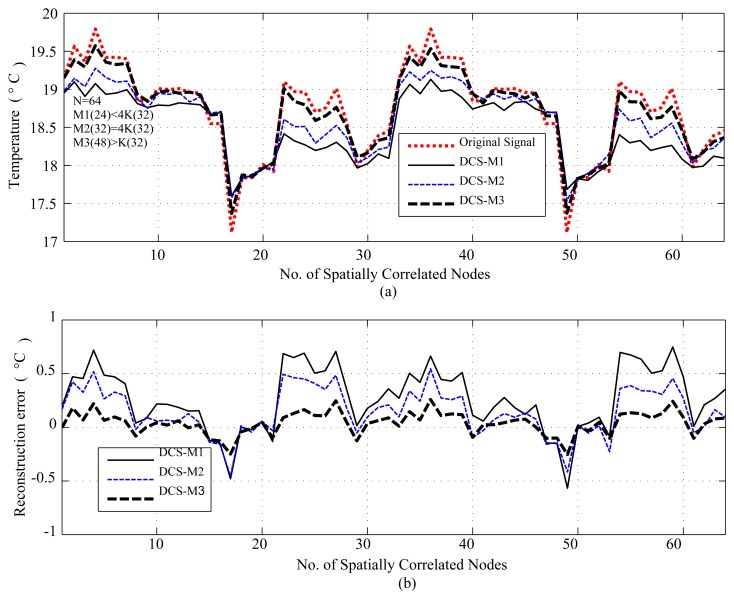
Distributed compressed sensing (DCS) in spatially correlated temperature data [94].

Figures 1, 2, 3, 4, 5, 6, 7 and 8 present the first part of the results. We present two results for each dataset and their corresponding data correlation (temporal or spatial). One for the sparsification or compressibility test and the other for the signal reconstruction, which visualize the potential of CS/DCS energy-efficient sampling in WSNs. The results of compressibility include the number of significant coefficients in wavelet analysis and their fit with the power law. For the reconstruction, we performed experiments for *N* = 1,024 and 2,048 for the temperature (temporal) and volcanic (temporal) datasets and *N* = 512 and 1,024 for CO_2_ (temporal) with variable *M*. Due to space limitations, we only present plots for *N* = 1,024 for temperature and volcanic datasets and *N* = 512 for CO_2_, but summarizing all of the results in a table.

As shown in Figures 1, 2 and 3, the considered temporally correlated temperature, seismic signal and CO_2_ and spatially correlated temperature signals are compressible as their discrete wavelet transform (DWT) analysis shows that the number of significant wavelet coefficients are very limited. It is clear from these figures that the sparsity levels of the datasets are diverse. For instance, the approximate number of significant coefficients (using balanced sparsity-norm thresholding) for temporally correlated temperature and CO_2_ are 39 (out of 2,048 in the figure, only 512 are shown) and 32 (out of 1,024 in figure, only 256 are shown), respectively, and for spatially correlated temperature it is eight (out of 64). On the other hand, using the same transform and thresholding, the approximate number of significant coefficients for a temporally correlated seismic wave are 49 (out of 1,024) and 177 (2,048), which are reasonably higher than the other two datasets. These are the values of *K* for the respective signals. Most importantly, these datasets are highly compressible, as their sorted (descending order) wavelet coefficients have good fit with the power law (shown in Figures 1 and 3), hence strongly satisfy the compressibility condition [13]. Even though all the datasets are compressible using CS/DCS, the compressibility of seismic wave might not be that significant. For instance, according to “four-to-one” [12], *M* = 4*K* for 2,048 samples (N) *M* = 4 * 177 = 708, which means M is almost comparable to *N*. One of the reasons for this could be the choice of transform or representation basis. The wavelet-based transforms are more suitable for piecewise constant data [26,88], but may not be for frequently variable seismic waves.

Figures 4, 5, 6, 7 and 8, present the results for signal reconstruction along with *R_mean_* (we have chosen *M* = 4*K*). Each figure presents the reconstruction result of a fixed *N* with four values of *M*, which are marked as CS-M1, CS-M2, CS-M3 and CS-M4. For temperature, the values of *M* were *M*1 = 90, *M*2 = 128, *M*3 = 256 and *M*4 = 512, for a seismic wave *M*1 = 256, *M*2 = 512, *M*3 = 768 and for CO_2_ *M*1 = 80, *M*2 = 128, *M*3 = 256 and *M*4 = 384. As shown in Figure 4, for the temporally correlated temperature data with samples *N* = 1,024 and *M*1 = 90, reconstruction slightly suffers with *R_mean_* = 0.39 and *R_max_* = 1.63 > 0.5°C [56] as *M*1(90) < 4*K*(100), where 4*K* is the standard required sample for satisfactory reconstruction. For the values of M close to 4*K* or higher, CS reconstruction perform satisfactorily as their *R_mean_* < 0.5 °C [56]. For instance, for *M*1 = 128, CS shows reasonably good performance with *RMSE* = 0.0173 with tolerable absolute residual mean (*R_mean_*) 0.23 °C, which is lower than the sensor tolerance, 0.5 °C [56]. As shown in Figure 4b, the reconstruction error or residual errors reduce progressively as *M* moves from lower to higher values. For example, in the case of *M*2 = 128, *R_mean_* = 0.23, and for *M*3 = 256, it is 0.082. Figure 5 presents the results for a temporally correlated CO_2_ signal with *N* = 512. Performance-wise, it shows similar trends as for Figure 4. For M1(80) < 4*K* (*88*), CS slightly suffers, with *R_mean_* = 1.71 and *R_max_* = 7.8, and for *M*2(128), *M*3(256) and *M*4(384), it shows satisfactory results for CO_2_ data, as their *R_mean_* values (e.g., 1.14, 0.49, 0.19) for all *M* are significantly lower than the typical CO_2_ sensor tolerance (e.g., ±40) [9]. This is because *M*(128/256/384) > 4*K*(88) [9]. Figure 6 presents the results for a temporally correlated seismic signal with *N* = 1,024. Although with increased *M*, it shows similar trends as for Figures 4 and 5, it suffers in terms of reconstruction quality, especially in terms of *R_mean_* and *RMSE*. For instance, for *M* = 256, *R_mean_* = 0.00055, comparable to the mean signal value, 0.0095, and *RMSE* = 0.72, which is really high compared to the other datasets. With the increased *M* for fixed *N*, both parameters improve with the reduced compression, but still suffer compared to the other datasets. This could be due to the inappropriate choice of the transform basis, as the used wavelet basis is good for piecewise constant data and may not be good for seismic wave-like continuously varying signals. This result shows the importance of the selection of an appropriate basis or transform, which is an important issue in CS/DCS.

Figures 4, 5 and 6 show the results for regular monitoring applications. Figure 7 presents the result of event detection using CS for a temporally correlated (temperature) signal. It is clear from the figure that CS has the potential to detect event (e.g., abrupt changes in temperature readings, which are available in dataset one) in a temporal signal with high accuracy (very low reconstruction error) and significant sensing compression (e.g., 50% for the figure where *N* = 1, 024 and *M* = 512).

Figure 8 presents the reconstruction result for spatially correlated signals for *N* = 64 where *K* = 8. As shown, for a lower value of measurements *M*1(24) < 4*K*(32) for *N* = 64, DCS reconstruction is poor, even in the case of *M*2(32) = 4*K*(32), due to a lower value of *N*. This is because CS/DCS has a scalability problem for lower values of *N*. On the other hand, *M*2 = 48 shows low *R_mean_* (0.093), but comes at the cost of reduced *SR* (1.34) compared to *SR* (2) for *M*2. It is evident from Figures 4, 5 and 6, 8, for values of *M* close to 4*K* or higher, CS reconstruction performs well for temporally and spatially correlated signals by providing *SR* > 1, hence sensing energy cost savings. Higher values of *M* progressively improve the reconstruction quality of CS/DCS, but higher values of *M* may be comparable to *N* and can diminish the advantages of CS/DCS.

### 5.2. Quantitative Analysis of Energy Cost Savings

For the second part of the analysis, we used Equation (9) to calculate sampling ratios (*SR*) and Equations (10) and (11) to calculate the corresponding sensing and overall energy savings (approximated) due to CS/DCS for each dataset. Here, $SR_{\text{eff}}=\frac{1}{SR}$ represents the fraction of the original samples that are needed (lower effective sampling rate = *SR_eff_* * original sampling rate) after compression to reconstruct the signal, and (1 – 1/*SR*)% is the saving in sensing. In CS/DCS, an effective sampling rate is always less than the Nyquist rate [13]. The overall energy savings due to CS and DCS were calculated based the information contained in Table 3, in [65,97]. The results are presented in Table 4. We disregarded the decoding cost, as the decoder (base station) in WSNs is normally main power connected.

As shown in Table 4, with the reduced sampling rate, CS can reduce *E_sm_* by 87.5%–25.37% (depending on *M*) for temperature and CO_2_ datasets; for the seismic dataset, it can be 75%–25.37% (depending on *M*). DCS can reduce *E_sm_* by 50%–25.37% (depending on *M*). In CS, the overall energy savings are almost similar to their *E_sm_* savings, as they need only a floating point operation, which costs very little compared to their sensing (*E_sm_*), communication (*E_comm_*) and computation (*E_comp_*) energy costs. As we have considered random sampling and correlation-based clustered WSNs, hence, in DCS, local communication costs in calculating *E_encoding_cs__* are disregarded. For similar values of *SR*, DCS suffers compared to CS in terms of *R_mean_*, due to lower values of *N*. It is clear from the trends in Table 4 that, for the same value of *M*, signals with higher values of *N* suffer in the reconstruction in terms of higher *R_mean_* (e.g., for *M* = 256, *R_mean_* is 0.082 for *N* = 1, 024 and 0.16 for *N* = 2, 048) and higher *RMSE*, as they have higher 4*K* requirements. Similarly, for fixed *N*, higher values of *M* show better performance by providing lower *R_mean_* and *RMSE*. This is clarified in Figures 4, 5 and 6, 8. For fixed *N*, a higher *M* means more measurements and a lower *SR*, hence better *R_mean_*. Depending on the application, a tradeoff between energy efficiency (*SR*), especially sensing energy efficiency, and *R_mean_* or *RMSE* might be needed. Even for similar *SR*, reconstruction with higher *N* shows better *SR* and *E_mean_*, as they have more measurements with which to play.

**Figure 9.**
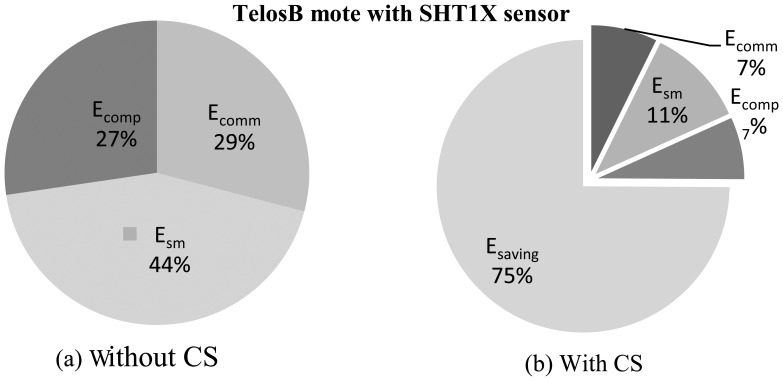
Comparison of *E_comm_*, *E_sm_* and *E_comp_*.

**Figure 10.**
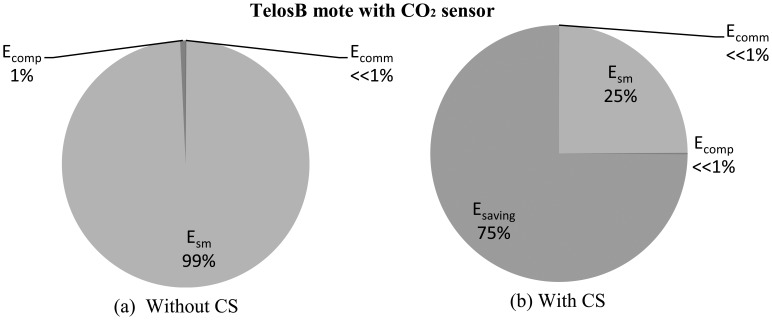
Comparison of *E_comm_*, *E_sm_* and *E_comp_*.

Figures 9 and 10 present comparison snapshots of *E_comm_*, *E_sm_* and *E_comp_*, normalized by *E_comm_* for temperature and CO_2_ sensors when attached to a TelosB mote [65] for *N* = 1,024 and *M* = 256 and *N* = 512 and *M* = 128, respectively. In summary, these figures and the Table 4, along with Figures 4, 5, 7 and 8, show the potential of CS and DCS in saving sensing and overall energy costs in WSNs. These benefits are coming at the cost of increased complexity at the sink and increased delay. This delay can be problematic in real-time WSN applications.

### 5.3. Comparative Study

**Figure 11.**
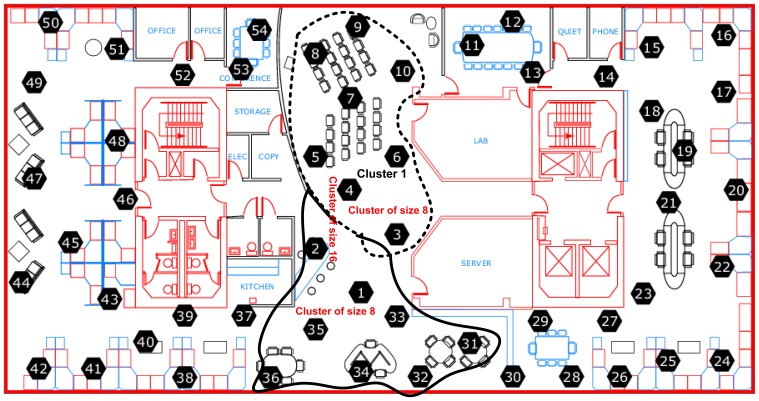
Intel-lab wireless sensor network (WSN) marked with sample clustering.

**Figure 12.**
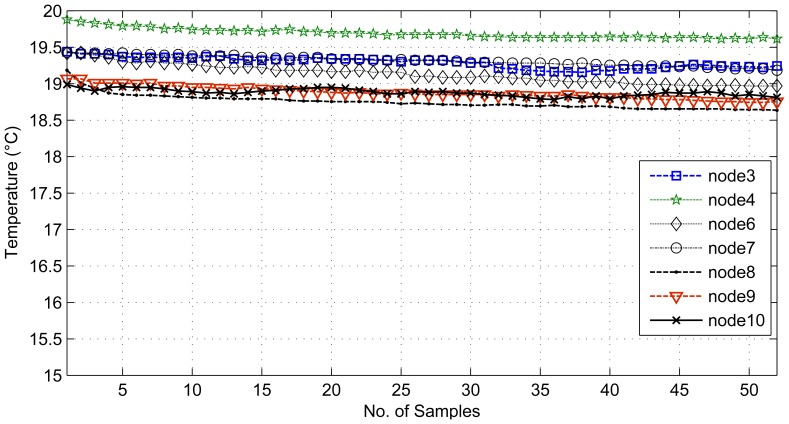
Spatial correlation in cluster 1 of Figure 11.

For the comparative study, we present two sets of results. The first set provides results for temporally correlated signals, where we compare CS, transform coding (TC), predictive coding with uniform sampling (PC-US) and adaptive sampling (PC-AS), and the second set for spatially correlated signals, where we compare DCS with TC and ASAP [36]. These results are mainly in terms of reconstruction performance and energy savings. Each set of results includes the performance for regular monitoring and event detection signal reconstructions. For spatially correlated sensor readings, we considered a clustered WSN and applied the schemes at the cluster level. It is quite evident from Figure 11 that the sensor readings of nodes 3, 4, 6, 7, 8, 9 and 10 are strongly correlated with the average correlation coefficient = +0.86. Therefore, similar to [36], we are assuming a correlation-based clustered WSN. In Figure 11, the thick dotted and continuous lines mark two clusters of size 8, and together, these two make a cluster of size 16. Figure 12 shows a snapshot of the spatial correlation amongst the nodes in cluster 1 of Figure 11.

For transform coding, as in CS and DCS sparsity analysis, we use the Haar wavelet transform. In particular, we exploit threshold-based transform coding, where transform coefficients under certain threshold values are discarded and others are sent to the sink, reducing communication cost. Balanced sparsity-norm thresholding-based two-level Haar wavelet transform [102] is used. For the temporally correlated sensor readings, each sensor collects readings over *n_s_* sampling periods and then applies transform [7,103,104] coding to determine the coefficients of each measurement, and after thresholding, the node sends the significant coefficients to the sink. In the case of spatially correlated readings, members of a cluster apply a level 1 transform and send their coefficients to the clusterhead, which applies a level 2 transform with the received readings and its own readings and sends the coefficients to the sink, which does the reconstruction. For simplicity, we do not consider any encoding of the transform coefficients [105].

In general, compressive sensing (CS) integrates the signal acquisition and compression steps into a single process [11–13]. Herein, we combine adaptive sampling [35] and an autoregressive-based prediction model [49,106] for temporally correlated readings or signals. Instead of the CUSUMtest, we use prediction error to detect non-stationarity changes in sensor readings. For spatially correlated readings, we use ASAP [36]. Here PC-AS and ASAP are the representatives of the down sampling method. As we are assuming a correlation-based clustered WSN for all the schemes, in the ASAP implementation, we consider only sub-clustering, sampler selection and the prediction model for non-sampler nodes. Selective sampling in ASAP contributes to sensing level compression and the prediction model to communication level. For the detail of these schemes, please see the [35,36,49,106]. The forms of information used in the ASAP implementation are: sampling fraction *σ* = 0.25, sub-cluster granularity *β* = 8, desired sampling period *τ_d_* =sampling period (dataset one), forced sampling period, *τ_f_*, and schedule update period, *τ_u_*, are based on prediction error.

**Figure 13.**
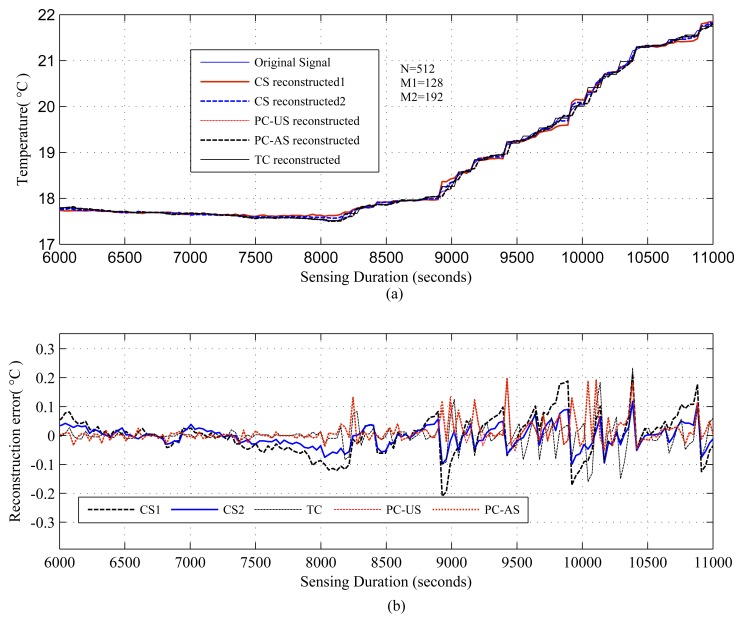
Comparison between CS, predictive coding with uniform sampling (PC-US), adaptive sampling (PC-AS) and transform coding (TC) for temperature signals.

**Figure 14.**
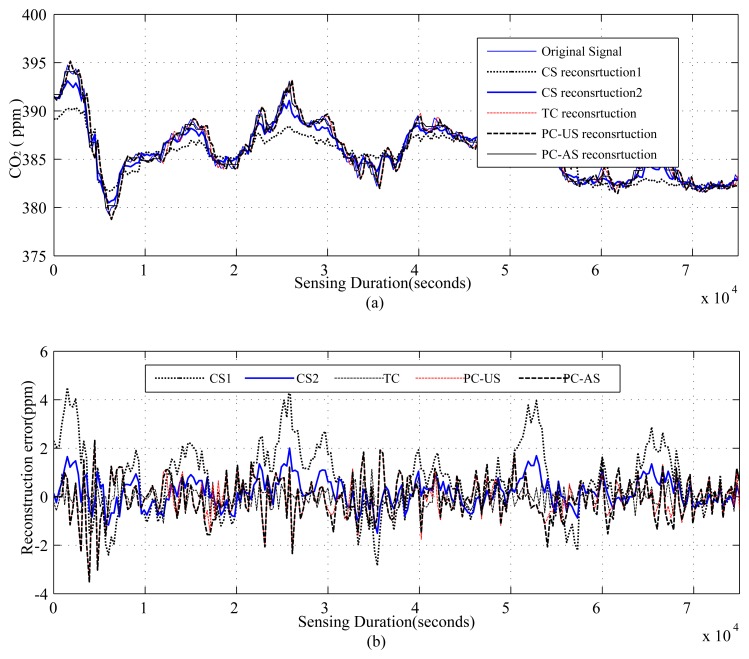
Comparison between CS, PC-US, PC-AS and TC for CO_2_ signals.

**Figure 15.**
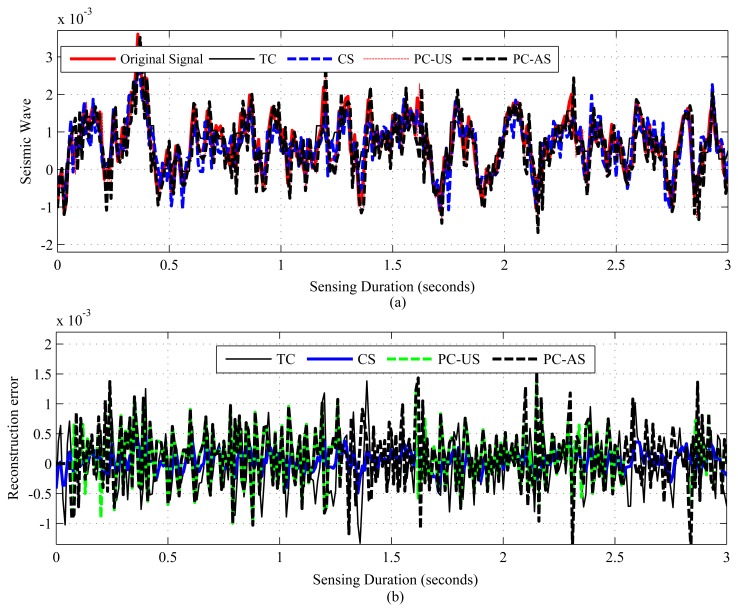
Comparison between CS, TC, PC-US and PC-AS for seismic signals.

**Figure 16.**
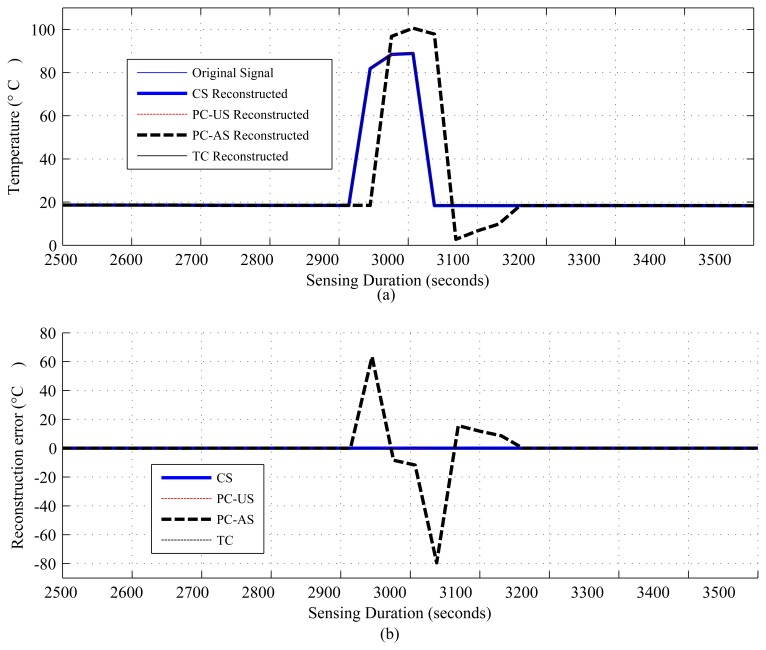
Comparison between CS, PC-US, PC-AS and TC in terms of event detection for a temporally correlated signal.

**Figure 17.**
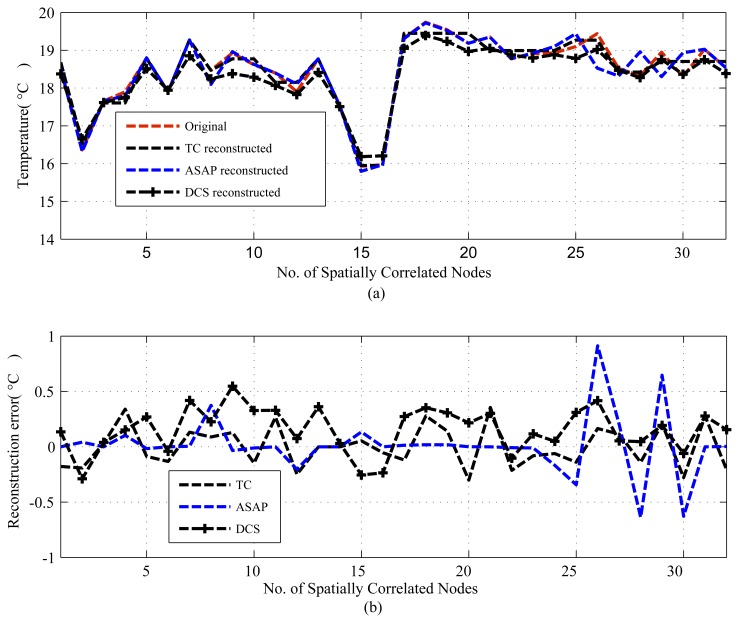
Comparison between DCS, the adaptive sampling approach (ASAP) and TC in a spatially correlated signal.

**Figure 18.**
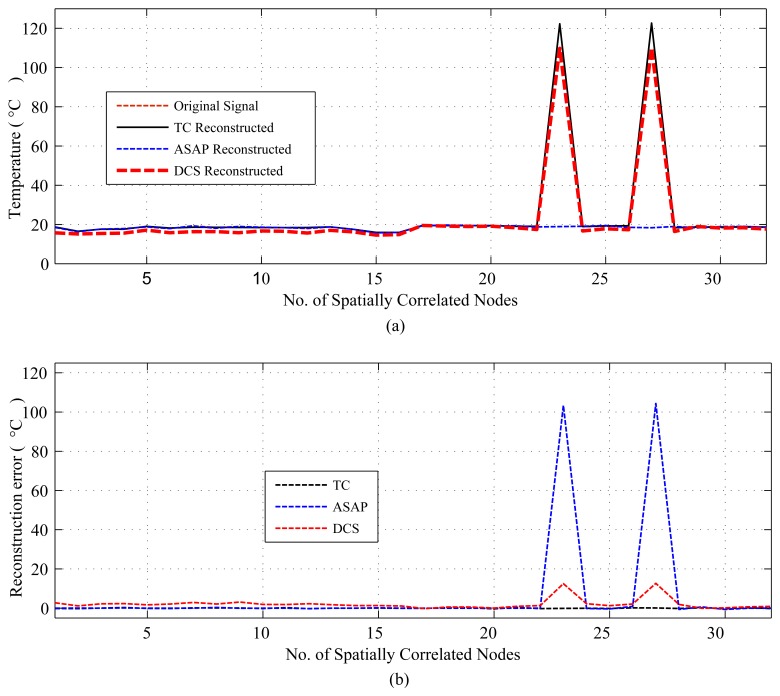
Comparison between DCS, ASAP and TC in terms of event detection for a spatially correlated signal.

Figures 13, 14, 15 and 16 present the results for the comparative study between CS,TC, PC-US and PC-AS, and Figures 17 and 18 present the results for the comparative study between DCS, TC and ASAP. We have used fixed *N* and two values of *M* in the temperature and CO_2_ datasets (mentioned in the figures), but one *M* in the volcanic dataset. The performance is summarized in terms of sensing energy minimized *E_sm_min__*, overall energy savings *E_saving_*, *R_mean_*, *RMSE* and event detection capability in Tables 5 and 6. CS using M1 (CS1) and PC-AS perform less well than TC and PC-US in terms of *R_mean_* and *RMSE*, but they provide better SR and, hence, better sensing and overall energy savings.

As Table 5 shows, in terms of *R_mean_* and *RMSE*, all schemes performing well above the sensor tolerances [9,56] in the temperature and CO_2_ datasets, but struggle in the seismic dataset. A possible reason for this struggling has been briefly mentioned earlier. For all the datasets, including the seismic one, CS outperforms all its counterparts in terms of sensing and overall energy savings. In the case of temperature signals, all schemes provide significant overall energy savings, but TC and PC-US perform poorly for CO_2_ signals, as the sensing cost of CO_2_ sensors is extremely high compared to others. Finally, in TC, CS and PC-US, event detection is always possible with good accuracy, but PC-AS is unreliable (Figure 16) since down-sampling might cause the event to be missed, as in the considered scenario.

For spatially correlated data, we performed experiments for *N* = 16 (DCS1) and 32 (DCS2). Due to space limitations, we only present plots for *N* = 32 or a cluster size of 32, but summarizing all of the results in Table 6. Figures 17 and 18 present the results for a cluster size of 32. As we can see from Table 6 and Figure 17, in terms of *R_mean_* and *RMSE*, all schemes perform reasonably well compared to the sensor tolerances [56]. In sensing and overall energy cost savings, DCS (DCS2) with a cluster size of 32, outperforms the other schemes. For the considered dataset, DCS and ASAP have highly comparable sensing-level energy savings, and ASAP performs less well in terms of overall energy cost savings, due to model learning and calculation. Most importantly, as shown in Figure 18, unlike CS and TC, ASAP might fail to detect events. This could be due to the correlation-based sub-clustering in ASAP. In ASAP, a sub-cluster can be comprised of nodes that are physically distant, and the selection of these distant nodes as non-sampler nodes on the basis of remaining power can cause events in proximity of those sensors to be missed.

Based on the results, tables and using information from [7], we summarize the results in Table 7. It is quite evident from this table and the above discussion that CS and DCS have the potential to sense energy efficiently and save overall energy costs. They can even can outperform most of their counterparts, especially down sampling methods, like PC-AS, ASAP, *etc.* However, delay can be an issue in real-time applications and in large-scale WSNs, and a lack of sparsity can be a problem in small WSNs. TC and PC-US perform less well than CS/DCS, PC-AS and ASAP, as they do not support sensing-level compression. For this reason, in power hungry sensors, e.g., CO_2_ sensors, communication and computational energy cost savings are almost nullified by high sensing costs. Due to the cost of model update and re-training, PC-US, PC-AS and ASAP might performs poorly in dynamic networks and environments where frequent updates are necessary. Hence, PC-AS and ASAP may fail to detect events (Figure 18). Moreover, ASAP performance depends on so many parameters [36] that it is hard to optimize and generalize for groups of applications.

## 6. Conclusion and Future Work

Most existing works on the energy management of WSNs disregard sensing energy cost, assuming that it is significantly less than that of sensor data communication. In this work, we have quantified the main operational energy costs in WSNs for some popular sensors, radios and sensor motes. The results presented in Table 3 clearly show that in a number of practical applications, the energy consumption of the sensing operation is comparable to, or even greater than, that of the radio. Cognizant of the importance of sensing energy costs, we have evaluated CS and DCS as potential approaches in energy-efficient sensing and overall energy cost savings in WSNs. To show the potential of CS and DCS in efficient sensing and overall energy cost savings, we have presented three sets of results. The first set clearly shows that temperature, seismic and CO_2_ signals are sparsely representable and, so, compressible, allowing CS and DCS to be effectively applied. The results also give the reconstruction accuracy of CS and DCS. The second set of results quantifies the potential of CS and DCS in saving sensing and overall energy costs. Finally, a comparative study between CS/DCS with their counterparts, especially down sampling methods (e.g., PC-AS, ASAP), was undertaken. This study clearly showed that CS and DCS are better schemes in terms of sensing and overall energy cost savings than TC, PC-US, PC-AS and ASAP. These results show that CS and DCS can save sensing and overall energy costs and can be used for energy-efficient data sensing and gathering in WSNs, especially in WSNs with energy hungry sensors.

The computational complexity of CS/DCS encoding is not significant, but decoding complexity (*O*(*n*^3^)) can be [69]. Due to decoding complexity, CS/DCS might not be suitable for real-time applications employing large WSNs. Investigation of decoding complexity reduction for CS/DCS is a recommended future research direction. In experiments, we considered clustered WSNs, which might be unavailable in some WSN applications. Investigations for other WSNs structures would be of merit.

## Figures and Tables

**Table 1.** Sensing energy of the sensors.

**Sensor Type**	**Representative Sensors**	***T****_s_***(*s*)**	***T****_r_***(*s*)**	***E****_sm_***(*mJ*)**
Acceleration	MMA7260Q [52]	0.001	0.002	0.0048
Pressure	2200/2600 Series [53]	NA	0.0005	0.0225
Light	ISL29002 [54]	NA	0.11	0.123
Proximity	CP18 [55]	0.1	0.001	48
Humidity	SHT1X [56]	0.011	8	72
Temperature	SHT1X [56]	0.011	5–30	270
Level	LUC-M10 [57]	NA	2	1,660
Gas(VOC)	MiCS-5521 [10]	30	30	4,800
Flow Control	FCS-GL1/2A4-AP8X-H1141 [58]	2	12	17,500
Gas (CO_2_)	GE/Telaire 6004 [9]	120–600	120	225,000

**Table 2.** Radios investigated.

**Components of *E****_comm_*	**CC2420 [61]**	**CC1000 [62]**	**AT86RF230 [63]**	**TDA5250 [64]**
*E_tx_*(*mJ*)	5.97	52.97	5.13	18.83
*E_rx_*(*mJ*)	6.38	19.62	4.83	97.3
*E_listen_*(*mJ*)	30.13	13.83	22.12	85.7
*E_slp_*(*mJ*)	1.077	1.078	6.47	0.0054
*E_sw_*(*mJ*)	136.54	194.4	172.73	669.6
*E_comm_*(*mJ*)	180.10	281.87	211.29	871.45

**Table 3.** Comparison of *E_comm_* with *E_sm_* and *E_comp_*.

**Sensors**	**TelosB**		**Mica2**		**Imote2**	

	***E****_sm_*	***E****_comp_*	***E****_sm_*	***E****_comp_*	***E****_sm_*	***E****_comp_*
MMA-7260Q	0.0000268	0.044	0.000017	0.096	0.0000268	4.01
2200/2600 Series	0.00013	0.044	0.000079	0.096	0.00013	4.01
ISL29002	0.00068	0.047	0.00044	0.106	0.00068	4.13
CP18	0.267	0.047	0.17	0.105	0.267	4.12
SHT1X (H)	0.4	0.043	0.255	0.77	0.4	12.8
SHT1X (T)	1.5	0.94	0.957	2.65	1.5	37
LUC-M10	9.22	0.104	5.89	0.266	9.22	6.2
MiCS-5521	26.98	1.84	17.242	5.2	26.98	69.9
FCS-GL1/2A4-AP8X-H1141	97.2	0.46	62.1	1.28	97.2	19.4
GE/Telaire 6004	1,249.25	9.03	798.2	25.64	1,249.25	333.8

**Table 4.** Numeric experiments: performances.

**Approach**	***N*/*K*/*M***	***SR***	***SR****_eff_*	***E****_smmin_*	***E****_saving_*	***R****_mean_*	***RMSE***
*CS_temp_*	1,024/25/128	8	0.125	87.5%	87.4%	.23	0.0173
*CS_temp_*	1,024/25/256	4	0.25	75%	74.9%	0.082	0.0068
*CS_temp_*	1,024/25/512	2	0.5	50%	49.9%	0.038	0.0039
*CS_temp_*	2,048/39/256	8	0.125	87.5%	87.4%	0.16	0.0133
*CS_temp_*	2,048/39/512	4	0.25	75%	74.9%	0.06	0.0051
*CS_temp_*	2,048/39/768	2.67	0.374	62.42 %	62.3%	0.03	0.0029
*CS_volc_*	1,024/49/256	4	0.25	75%	74.88%	0.00055	0.72
*CS_volc_*	1,024/49/512	2	0.5	50%	49.89%	0.00031	0.50
*CS_volc_*	1,024/49/768	1.34	0.746	25.37%	25.21%	0.000153	0.311
*CS_volc_*	2,048/177/768	2.67	0.374	62.42%	62.3%	0.00054	0.35
*CS_volc_*	2,048/177/1,024	2	0.5	50%	49.89%	0.00039	0.28
*CS_volc_*	2,048/177/1,536	1.34	0.746	25.37%	25.21%	0.00019	0.16
*CS_CO_*_2_	512/22/128	4	0.25	75%	74.9%	1.14	0.0054
*CS_CO_*_2_	512/22/256	2	0.5	50%	49.9%	0.49	0.0028
*CS_CO_*_2_	512/22/384	1.34	0.746	25.37%	25.2%	0.19	0.0015
*CS_CO_*_2_	1,024/32/128	8	0.125	87.5%	87.4%	1.63	0.0072
*CS_CO_*_2_	1,024/32/256	4	0.25	75%	74.9%	0.93	0.0046
*CS_CO_*_2_	1,024/32/512	2	0.5	50%	49.9%	0.4	0.0023
*DCS_temp_*	32/6/16	2	0.5	50%	49.9%	0.39	0.033
*DCS_temp_*	32/6/24	1.34	0.746	25.37%	25.2%	0.104	0.014
*DCS_temp_*	64/8/32	2	0.5	50%	49.9%	0.2	0.0235
*DCS_temp_*	64/8/48	1.34	0.746	25.37%	25.2%	0.093	0.014

**Table 5.** Numeric experiment: comparative study with temporally correlated signals.

**Approach**	***SR***	***E****_smmin_*	***E****_saving_*	***R****_mean_*	***RMSE***	**Event Detection**
*CS*1*_temp_*	4	75%	74.9%	0.06	0.0051	Possible
*CS*2*_temp_*	2.67	62.54%	62.43%	0.05	0.0045	Possible
*TC_temp_*	1	0%	34.3%	0.022	0.0018	Possible
*PC* – *U S_temp_*	1	0%	26.05%	0.0214	0.0016	Possible
*PC* – *AS_temp_*	1.14	12.5%	31.23%	0.0218	0.0016	Not always
*CS*1*_CO_*_2_	4	75%	74.9%	1.29	0.0028	Possible
*CS*2*_CO_*_2_	2	50%	49.4%	0.5	0.0032	Possible
*TC_CO_*_2_	1	0%	0.06%	0.37	0.0012	Possible
*PC* – *US_CO_*_2_	1	0%	0.036%	0.64	0.0017	Possible
*PC* – *AS_CO_*_2_	1.21	17.41%	37.39%	0.67	0.0036	Not always
*CS_volc_*	1.34	25.37%	25.21%	0.000145	0.325	Possible
*TC_volc_*	1	0%	20.06%	0.00035	0.3825	Possible
*PC* – *U S_volc_*	1	0%	20.036%	0.000415	1.79	Possible
*PC* – *AS_volc_*	1.041	4.15%	23.39%	0.00045	1.89	Not always

**Table 6.** Numeric experiments: comparative study with spatially correlated signals.

**Approach**	***SR***	***E****_smmin_*	***E****_saving_*	***R****_mean_*	***RMSE***	**Event Detection**
*DCS*1*_temp_*	1.46	31.5%	31.45%	0.21	0.028	Possible
*DCS*2*_temp_*	2	50%	49.3%	0.2	0.023	Possible
*TC*1*_temp_*	1	0%	38.2%	0.16	0.012	Possible
*TC*2*_temp_*	1	0%	38.2%	0.14	0.010	Possible
*ASAP*1*_temp_*	1.59	37.1%	36.5%	0.15	0.0145	Not always
*ASAP*2*_temp_*	1.45	31.2%	30.7%	0.13	0.0139	Not always

**Table 7.** Comparative summary of the considered schemes.

**Issues**	**CS**/**DCS**	**TC**	**PC**-**US**	**PC**-**AS**	**ASAP**
Complexity	*O*(*M*)	*O*(*N*)	*O*(*m*^3^*n_ls_*) ^1^	*O*(*m*^3^*n_ls_*)	*O*(*m*^3^*n_ls_*)
*E_sm_min__*	significant	no	no	moderate	significant
*E_saving_*	significant	not always	not always	moderate	significant
*R_mean_*	low	low	low	low	low
*RMSE*	low	low	low	low	low
Delay	could be high	moderate	low	low	low
Event Detection	possible	possible	possible	not always	not always
Scalability	medium	low	low	low	medium

1 Where *m* is the order of the model and *n_ls_* is the learning samples.
